# Anti-Obesity Effect of Standardized Extract of Microalga *Phaeodactylum tricornutum* Containing Fucoxanthin

**DOI:** 10.3390/md17050311

**Published:** 2019-05-27

**Authors:** Song Yi Koo, Ji-Hyun Hwang, Seung-Hoon Yang, Jae-In Um, Kwang Won Hong, Kyungsu Kang, Cheol-Ho Pan, Keum Taek Hwang, Sang Min Kim

**Affiliations:** 1 Natural Product Informatics Center, KIST Gangneung Institute of Natural Products, Gangneung 25451, Korea; ninesong2@kist.re.kr (S.Y.K.); hwangjh@kist.re.kr (J.-H.H.); kskang@kist.re.kr (K.K.); panc@kist.re.kr (C.-H.P.); 2 Smart Farm Research Center, KIST Gangneung Institute of Natural Products, Gangneung 25451, Korea; 3 Department of Medical Biotechnology, College of Life Science and Biotechnology, Dongguk University, Seoul 04620, Korea; shyang@dongguk.edu; 4 R&D Department, AlgaeTech Co. Ltd., Gangneung 25457, Korea; korea4633@naver.com (J.-I.U.); hongtol2@gmail.com (K.W.H.); 5 Department of Food and Nutrition, and Research Institute of Human Ecology, Seoul National University, Seoul 08826, Korea; keum@snu.ac.kr; 6 Division of Bio-Medical Science & Technology, University of Science & Technology, Daejeon 34113, Korea

**Keywords:** anti-obesity effect, *Phaeodactylum tricornutum*, fucoxanthin, *Phaeodactylum* extract, microalgae

## Abstract

Fucoxanthin (FX), a marine carotenoid found in macroalgae and microalgae, exhibits several beneficial effects to health. The anti-obesity activity of FX is well documented, but FX has not been mass-produced or applied extensively or commercially because of limited availability of raw materials and complex extraction techniques. In this study, we investigated the anti-obesity effect of standardized FX powder (*Phaeodactylum* extract (PE)) developed from microalga *Phaeodactylum tricornutum* as a commercial functional food. The effects of PE on adipogenesis inhibition in 3T3-L1 adipocytes and anti-obesity in high-fat diet (HFD)-fed C57BL/6J mice were evaluated. PE and FX dose-dependently decreased intracellular lipid contents in adipocytes without cytotoxicity. In HFD-fed obese mice, PE supplementation for six weeks decreased body weight, organ weight, and adipocyte size. In the serum parameter analysis, the PE-treated groups showed attenuation of lipid metabolism dysfunction and liver damage induced by HFD. In the liver, uncoupling protein-1 (UCP1) upregulation and peroxisome proliferator activated receptor γ (PPARγ) downregulation were detected in the PE-treated groups. Additionally, micro computed tomography revealed lower fat accumulation in PE-treated groups compared to that in the HFD group. These results indicate that PE exerts anti-obesity effects by inhibiting adipocytic lipogenesis, inducing fat mass reduction and decreasing intracellular lipid content, adipocyte size, and adipose weight.

## 1. Introduction

Fucoxanthin (FX), a marine xanthophyll carotenoid, is abundantly in macroalgae, such as *Laminaria japonica* and *Undaria pinnatifida* [1]. Recently, some microalgae including *Phaeodactylum tricornutum*, *Odontella aurita*, and *Isochrysis galbana* were reported as new sources for FX production [2,3,4]. FX has multiple health-promoting effects, such as antioxidant, anticancer, anti-inflammatory, and anti-obesity effects [5,6,7]. The anti-obesity effect is the most remarkable property and has been supported by numerous in vitro and in vivo studies [8,9]. FX exerts its anti-obesity effects through several mechanisms, which are influenced by numerous factors, including nutritional, hormonal, and genetic elements [10]. FX significantly reduces triglyceride concentrations in the liver and adipose tissue and beneficially influences cholesterol-regulating enzymes [11]. FX also affects gene expression associated with lipid metabolism, such as hepatic acetyl-CoA carboxylase, sterol regulatory element-binding protein, fatty acid synthase, stearoyl-coenzyme A desaturase-1, CCAAT/enhancer-binding protein α (C/EBPα), and peroxisome proliferator activated receptor (PPAR) α and γ [12,13]. Recently, the anti-obesity effect of FX was found to involve stimulation of the uncoupling protein-1 (UCP1) expression in white adipose tissue (WAT) [14,15,16]. Increased expression of mitochondrial UCP1 leads to increased energy expenditure. UCP1 is found in brown adipose tissue and represents a crucial factor in thermogenesis, which is heat production contributing to the reduction of WAT and physiological defense against obesity [17,18,19]. Additionally, the hormone, leptin, is mainly expressed in differentiated adipocytes of white tissue to maintain homeostatic regulation of the adipose tissue and body weight by controlling food intake and energy expenditure [20]. Many previous studies suggested that FX alters plasma leptin levels to achieve anti-obesity effects [21]. Many studies reported that FX supplementation derived from brown seaweed (macroalgae) clearly reduced WAT in rats and mice and improved weight-loss effects as well as plasma and hepatic lipid metabolism [22,23]. Numerous in vivo studies have been conducted to evaluate seaweed-derived FX, while few in vivo tests have used microalgae-derived FX. 

*Phaeodactylum tricornutum* (PT) is known as a potential source of FX production [2]. In PT, FX production is at least 10-fold higher than that in macroalgae. Therefore, PT may be valuable for treating and preventing obesity. PT biomass itself has been studied for its anti-obesity effect, but no studies have examined standardized materials containing FX extracted from marine microalgae as a health functional food, and no studies have evaluated the anti-obesity effects of these materials [24]. 

The aim of the present study was to evaluate the anti-obesity effect of the standardized extract of PT (*Phaeodactylum* extract, PE) containing 3.5~6% FX (*w/w*) on lipid accumulation in 3T3-L1 adipocytes and high-fat diet-induced obese mice. From in vitro and in vivo studies, we confirmed that PE exerted a strong anti-obesity effect by decreasing body weight, organ weight, and adipocyte size. In addition, UCP1 upregulation and PPARα downregulation were also observed in both studies, indicating the mechanism through which PE regulates lipid metabolism. Therefore, we concluded that PE, a new FX source produced from microalga PT, might be a potent anti-obesity material applicable to commercial functional foods.

## 2. Results

### 2.1. PE Reduced Lipid Accumulation in Adipocytes

PE is the standardized FX powder, bright red in color, which was produced from the microalga PT for commercial purposes. AlgaeTech Co. Ltd. (Gangneung, Korea) supplied FX content information of 3.5~6% (*w/w*) in PE. In order to quantify FX in PE, HPLC analysis was performed, as described in our previous report, with slight modification [2]. The result demonstrated that the FX peak in PE showed the same UV-VIS absorption spectrum with the FX standard (Figure S1A) and the average amount of FX was 47.74 ± 0.04 mg/g in three independent PE batches (Figure S1B). This indicated that the FX contents in the PE batches used in this study falls within the standardization range.

Prior to evaluating the effects of FX and PE in 3T3-L1 cells, we performed a cytotoxicity assay to determine the proper concentration of FX and PE for further analysis. We indicated that FX was not toxic to cells below 80 μM, and PE showed cytotoxicity at 1000 μg/mL (Figure S2). Therefore, we conducted experiments by using non-toxic concentrations of FX and PE. 

**Figure 1:**
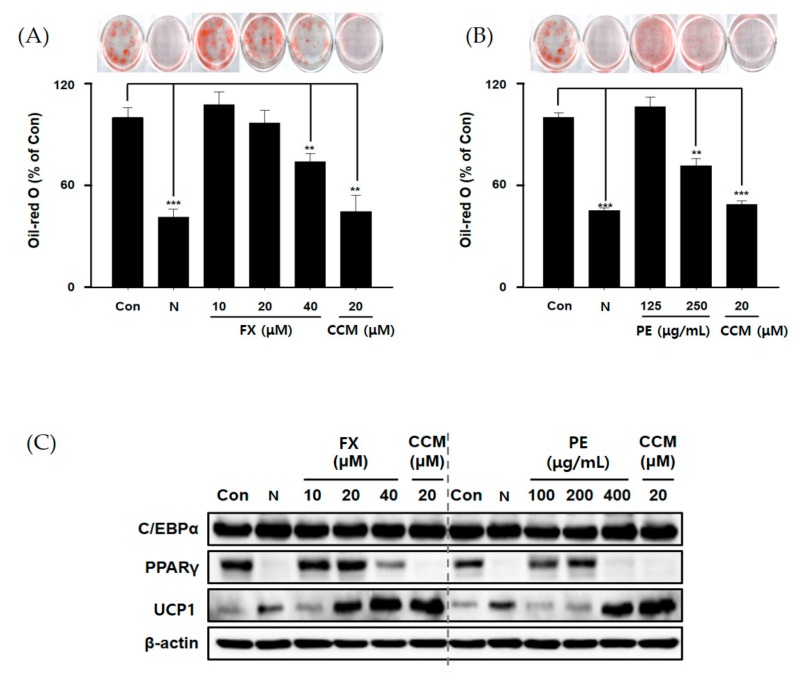
Effect of fucoxanthin (FX) and *Phaeodactylum* extract (PE) on lipid accumulation in 3T3-L1 cells during adipogenesis. Cells were cultured during differentiation (for six days) with FX, PE, or curcumin (CCM; reference). Accumulated lipids were stained with Oil-red O reagent and quantified by measuring the absorbance at 500 nm. (**A**) FX or CCM suppressed lipid accumulation; (**B**) PE and CCM inhibited lipid accumulation; (**C**) expression of proteins related to lipid accumulation (CCAAT/enhancer-binding protein α (C/EBPα), peroxisome proliferator activated receptor γ (PPARγ), uncoupling protein-1 (UCP1)). The experiment was performed in triplicate. ***/**/* indicate significant differences at *p <* 0.001/ *p <* 0.01/*p <* 0.05 compared to the control (differentiation). N indicates no differentiation.

To determine the effect of FX and PE on lipid accumulation, the cells were cultured during differentiation (for six days) with FX, PE, and curcumin (CCM; reference). Staining with Oil-red O (ORO), which is commonly used to detect intracytoplasmic lipid accumulation, demonstrated that FX causes a dose-dependent reduction in lipid accumulation. FX at 40 μM inhibited lipid accumulation by 30% compared to the control during differentiation. PE decreased lipid accumulation at 250 μg/mL during adipocyte differentiation (Figure 1A,B). Thus, FX and PE alleviated the effect of lipid accumulation on insulin-stimulated 3T3-L1 cells. 

Adipogenic differentiation is related to various adipogenic factors, such as C/EBPα, PPARγ, and UCP1, as mentioned above [12,13,14,15,16]. To determine whether these factors are controlled by FX and PE, we analyzed protein levels in the absence or presence of FX or PE during insulin-induced differentiation. At high levels, FX and PE clearly decreased PPARγ levels and dose-dependently increased the UCP1 level (Figure 1C). However, neither FX nor PE affected the level of C/EBPα. FX was reported to inhibit the intercellular lipid accumulation by reducing the expression of PPARγ and C/EBPα. In the case of C/EBPα, there have been some conflicting results about the effect of FX on the expression of this enzyme during the differentiation period [10,25,26]. In this study, we conclude that FX and PE treatment induced downregulation of PPARγ and upregulation of UCP1 in two key major enzymes (PPARγ and UCP1) in 3T3-L1 cells. We have also shown that FX, a major component of PE, can be one of the main ingredients for the anti-obesity effect of PE. Thus, it was concluded that PE has an anti-obesity effect by controlling lipid metabolism through PPARγ and UCP1.

### 2.2. Effect of PE on Body Weight, Liver, and Inguinal Fat Weights

**Figure 2:**
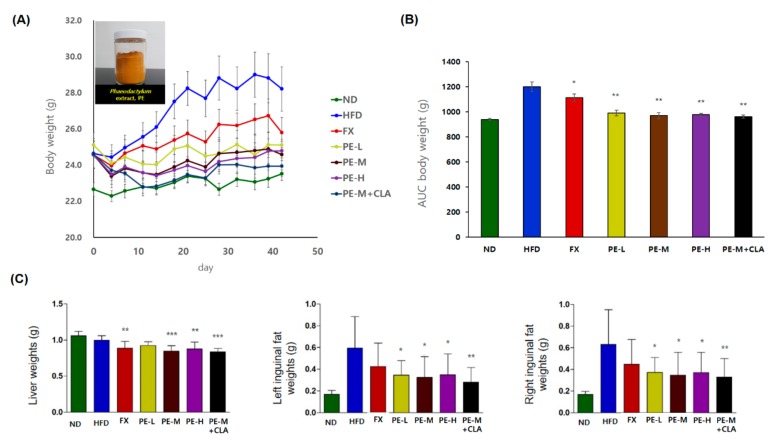
(**A**) Changes in body weight of C57BL/6J mice fed PE, (**B**) area under the curve data for body weight, (**C**) effects of PE on liver and inguinal fat weights in high-fat fed C57BL/6J mice. The groups are abbreviated as: Normal diet (ND); high-fat diet (HFD); fucoxanthin (FX), HFD + FX 0.1 mg/kg/day; PE-L, HFD + PE 0.81 mg/kg/day; PE-M, HFD + PE 1.62 mg/kg/day; PE-H, HFD + PE 3.25 mg/kg/day; PE-M + conjugated linoleic acid (CLA), HFD + PE 1.62 mg/kg/day plus CLA 410 mg/kg/day. ***/**/* indicate significant differences at *p <* 0.001/*p <* 0.01/*p <* 0.05 level compared to HFD. Values are shown as the mean ± SD (*n* = 10 per group).

Before treatment, the average initial body weight of the mice was 24.38 g, with no significant differences among the 7 groups. As shown in Figure 2A,B, the high-fat diet (HFD) group showed a large increase in body weight during the experimental period. After 6 weeks, the area under the curve (AUC) of body weights was higher in the HFD group than in the normal diet (ND) group and other groups (*p <* 0.001). Mice in the HFD plus PE treatment group showed a lower AUC level than the HFD group, while mice in the PE plus conjugated linoleic acid (CLA) group showed the lowest AUC level. For body weight gain, all PE-treated groups showed weight loss in a dose-dependent manner compared to the HFD group, with greater effects than in the positive control (FX). As shown in Figure 2C, we weighed the livers and inguinal adipose tissue. As a result, of liver weight measurement, PE treatment groups (PE-L, PE-M, PE-H) and the PE-M plus CLA group showed significantly lower weight values (1.12%~7.45% at *p <* 0.001 or *p <* 0.01) than in the HFD group. Inguinal adipose tissue weight was significantly higher in the HFD group than the ND group on both the right and left. However, the PE-treated groups exhibited lower values than the HFD group in inguinal adipose tissue weight over 40% (*p <* 0.01 or *p <* 0.05). Based on this result, we concluded that PE exerts anti-obesity effects in vivo in mice.

### 2.3. Effect of PE on Serum Lipid Parameters

Next, we measured serum parameters to evaluate the effect of PE on lipid metabolism as well as liver damage. As shown in Table 1, the HFD group showed a slight increase in the level of triacylglyceride (TG) and a significant increase in total cholesterol (TC) and low-density lipoprotein (LDL) compare with the ND group. These data demonstrated that HFD consumption induces lipid dysmetabolism. Administration of PE-M and PE-M + CLA for 6 weeks significantly decreased the TG level compared with that in the HFD group, while groups treated with FX, PE-L, and PE-H showed a slight decrease in the TG level compared with that of the HFD group. Neither FX nor PE affected the HFD-induced increase in TC and high-density lipoprotein (HDL) levels. FX treatment did not affect the levels of LDL, which is generally considered as a pathological marker. In contrast, PE administration decreased the LDL levels in a dose-dependent manner, especially PE-H, which significantly decreased the LDL levels. In addition, the HFD group also showed an abnormal increase in alanine aminotransferase (ALT) levels and a significant increase in aspartate aminotransferase (AST) levels compared with those in the ND group, indicating that HFD also induced liver injury [27]. The FX, PE, and PE-M + CLA groups showed a slight decrease in AST levels compared with the HFD group, although the difference was not statistically significant. FX and PE-M + CLA treatment significantly decreased ALT levels, whereas PE treatment slightly decreased ALT levels, compared with those in the HFD group. Based on these data, we concluded that PE-M, PE-H, and PE-M + CLA could attenuate lipid metabolism dysfunction and liver damage induced by HFD.

### 2.4. Effect of PE on Fat Accumulation

#### 2.4.1. Histological Analysis of the Liver

**Figure 3:**
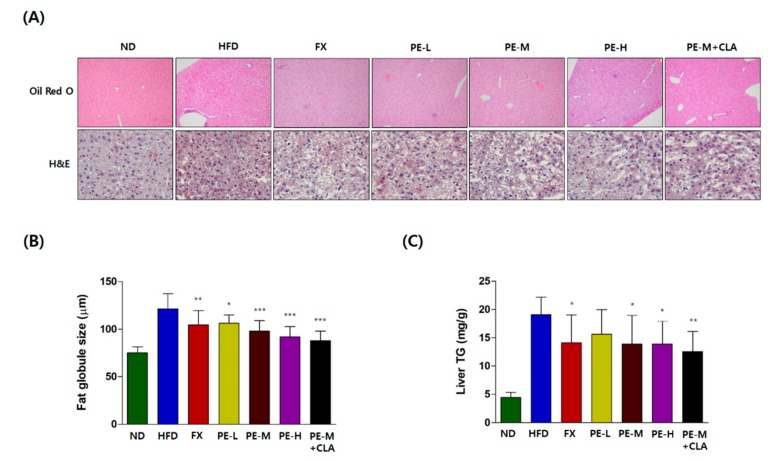
(**A**) Histological analysis of liver tissues. Liver tissue section stained with Oil Red O and hematoxylin and eosin (H&E) (magnification, ×100 (Oil Red O), ×400 (H&E)), (**B**) fat globule size, (**C**) liver TG. The groups are abbreviated as: Normal diet (ND); high-fat diet (HFD); fucoxanthin (FX), HFD + FX 0.1 mg/kg/day; PE-L, HFD + PE 0.81 mg/kg/day; PE-M, HFD + PE 1.62 mg/kg/day; PE-H, HFD + PE 3.25 mg/kg/day; PE-M + CLA, HFD + PE 1.62 mg/kg/day plus CLA 410 mg/kg/day. Lipid droplet numbers and fat globule size in the Oil Red O staining were dose-dependently decreased by PE treatment (**A**,**B**) and there was no significant difference in the cell staining pattern between the HFD and PE treatment groups in the H&E staining (**A**). ***/**/* indicate significant differences at *p <* 0.001/*p <* 0.01/*p <* 0.05 level compared to the HFD group. Values are shown as the mean ± SD (*n* = 10 per group).

For histological analysis, liver tissues were stained with ORO and hematoxylin and eosin (H&E) to observe the nucleus and cytoplasm of the cell [28]. As shown in Figure 3A, there was no significant difference in the cell staining pattern between the HFD and PE treatment groups in the H&E staining, indicating that the cell states of all groups were normal. However, ORO staining revealed that the livers of the HFD groups contained more lipid droplets than those of the ND group and the FX- and PE-treated groups with the increased fat globule size (Figure 3A). After treatment with FX or PE, the lipid droplet numbers decreased (Figure 3A) and the fat globule sizes reduced strongly to 75.66% of that in the HFD group in a dose-dependent manner (Figure 3B). In addition, the PE-M + CLA group showed the biggest reduction in fat globule size (72.5% of that in the HFD group, *p <* 0.001) indicating the synergistic effect of CLA on liver fat metabolism. In Figure 3C, the level of liver in TG in the PE-treated group reduced significantly and dose-dependently. These results clearly demonstrated the effect of PE on hepatic steatosis.

#### 2.4.2. Micro Computed Tomography Analysis

**Figure 4:**
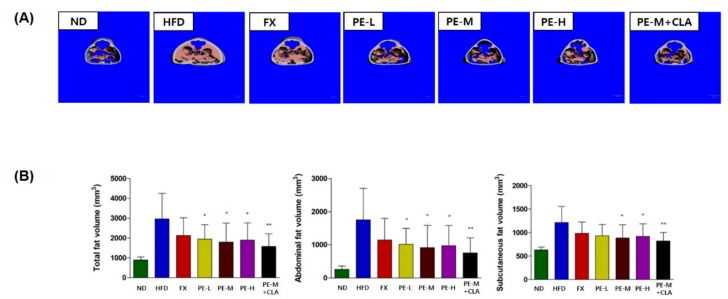
(**A**) Micro computed tomography (CT) analysis, (**B**) total, abdominal, and subcutaneous fat volumes. The groups are abbreviated as: Normal diet (ND); high-fat diet (HFD); fucoxanthin (FX), HFD + FX 0.1 mg/kg/day; PE-L, HFD + PE 0.81 mg/kg/day; PE-M, HFD + PE 1.62 mg/kg/day; PE-H, HFD + PE 3.25 mg/kg/day; PE-M + CLA, HFD + PE 1.62 mg/kg/day plus CLA 410 mg/kg/day. ***/**/* indicate significant differences at *p <* 0.001/*p <* 0.01/*p <* 0.05 levels compared to the HFD group. Values are shown as the mean ± SD (*n* = 10 per group).

The abdominal and subcutaneous fat in each mouse was assessed by micro computed tomography (CT) analysis. As shown in Figure 4A, fat accumulation was greater in the HFD group than in any other groups. PE-treated groups (PE-L, PE-M, PE-H, and PE-M + CLA) showed less distributed fat accumulation than the HFD group and less fat than in the positive control, FX. As shown in Figure 4B, total, abdominal, and subcutaneous fat volumes were evaluated and quantified by micro CT analysis. The results showed that all groups (HFD, FX, PE-L, PE-M, PE-H, and PE-M + CLA) were significantly different compared to the ND group (statistical data not shown). Additionally, the PE-treated groups (PE-L, PE-M, PE-H, and PE-M + CLA) showed decreased fat contents compared to the HFD group (*p <* 0.01 to < 0.05), and the fat content was the lowest in the PE-M + CLA combination group (*p <* 0.01). The total fat volumes in the PE-treated groups were 53.30~65.66% of that in the HFD group. For abdominal fat, the values in the PE-treated groups were 43.34~57.96% of that in the HFD, whereas for subcutaneous fat, the values in the PE-treated groups were 67.68~76.78% of that of the HFD group. These results suggest that PE inhibits adipocytic lipogenesis and reduces fat levels. These are consistent with those of weight loss.

### 2.5. PE Regulated the Expression Proteins Related to Lipid Metabolism in Adipose Tissue

**Figure 5:**
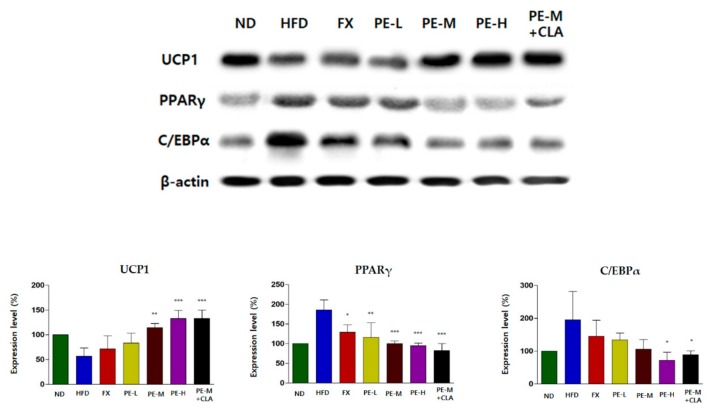
Effect of PE on the relative expression of lipid metabolism-related proteins in the liver. All expression levels (%) of each group were relatively compared with that of ND group (100%). The groups are abbreviated as: Normal diet (ND); high-fat diet (HFD); fucoxanthin (FX), HFD + FX 0.1 mg/kg/day; PE-L, HFD + PE 0.81 mg/kg/day; PE-M, HFD + PE 1.62 mg/kg/day; PE-H, HFD + PE 3.25 mg/kg/day; PE-M + CLA, HFD + PE 1.62 mg/kg/day plus CLA 410 mg/kg/day. ***/**/* indicate significant differences at *p <* 0.001/*p <* 0.01/*p <* 0.05 levels compared with HFD. Values are shown as the mean ± SD (*n* = 3 per group).

We assessed the expression of UCP1, PPARγ, and C/EBPα, which are related to lipid metabolism in adipose tissue. As shown in Figure 5, UCP1 expression was increased in the PE-treated groups. Particularly, compared to in the HFD group, UCP1 expression levels in the PE-M, PE-H, and PE-M + CLA groups were 2.02-, 2.34-, and 2.35-fold higher, respectively, indicating significant and dose-dependent effects. PPARγ expression levels were significantly lower in the PE-L, PE-M, PE-H, and PE-M + CLA groups than in the HFD group by 0.62-, 0.54-, 0.51-, and 0.45- fold, respectively, with high significance (*p < 0.001* or *p < 0.01*). In addition, C/EBPα expression levels were lower in the PE-treated groups. Significance was low (*p < 0.05)*, which is similar to the in vitro result in Figure 1. Overall, these results suggest that PE can upregulate UCP1, which is related to energy expenditure, and downregulate PPARγ, which is related to adipocyte differentiation.

## 3. Discussion

Application of microalgae has extended from biomass production for biodiesel to value-added products [29,30], and FX is one of the most prominent value-added products obtained from microalgae. However, to date, FX has been produced mostly by macroalgae, such as *L. japonica*, *U. pinnatifida*, and *Eisenia bicyclis* [1]. Numerous commercial products containing FX show anti-obesity activity; xanthigen is among the most representative products and contains 3 mg of FX for daily intake. However, FX has not been mass-produced or applied extensively in the global market. The first reason is the difficulty of extracting highly concentrated FX from the materials. Macroalgae contains polysaccharides, such as fucoidan and chlorophylls. These components make it difficult to purify FX after extraction, using solvents such as ethanol and acetone. Although several studies have attempted specific FX extraction or have used discarded parts of macroalgae [31,32], no economical method is available for producing FX with high purity. Additionally, macroalgae contains a low amount of FX. As reported in our previous study, the FX content is typically below 0.5% in fresh material [2,3], making solvent extraction difficult and reducing the economic feasibility of FX purification using simple solvents. Thus, solvent-extracted seaweed is used as an FX source in many commercial FX products available on the market but is not applied extensively in other fields, such as in food, cosmetic, and pharmaceutical products. Finally, seaweed has an unpleasant odor when extracted using simple solvents. Seaweed extract contains high concentration of chlorophylls and sticky materials. These components give seaweed a specific odor and dark color, making it difficult to apply in other industrial fields. 

In this study, we used the standardized FX powder (PE) from microalga PT. The amount of FX in PE ranged from 3.5% to 6% (*w/w*), and the sample was bright red in color and contained trace amounts of chlorophylls. This material can be utilized as a global source of FX like the global antioxidant carotenoid astaxanthin powder from the microalga *Haematococcus pluvialis*, which contains generally over 5% (*w/w*) astaxanthin [33]. Here, we evaluated the anti-obesity activity of this new standardized FX powder in vitro as well as in vivo using 3T3-L1 cells and C57BL/6J mice. The results showed that PE decreased lipid accumulation in 3T3-L1 cells in a dose-dependent manner. We confirmed that FX and PE alleviated lipid accumulation in adipocytes. Additionally, regarding adipogenetic factors, FX and PE downregulated PPARγ protein levels at high concentrations and upregulated the UCP1 protein level in a dose-dependent manner, but did not influence the C/EBPα protein level. In obese mice fed an HFD, six weeks of supplementation with PE decreased the body weight, organ weight, fat volume, and adipocyte size without affecting food intake (Table S1). Simultaneously, serum parameters, such as TC, TG, HDL, LDL, AST, and ALT were decreased, indicating attenuation of lipid metabolism dysfunction and liver damage induced by HFD. 

Hepatic steatosis, defined as induced excessive lipid accumulation in the liver, is highly related to obesity and other diseases [34]. In this study, PE effectively reduced the fat globule size compared to the HFD group, indicating that the enhanced anti-obesity effect of PE on hepatic steatosis was achieved mainly by lowering excessive fat accumulation (Figure 3). Additionally, the level of liver TG was significantly reduced in a dose-dependent manner, indicating decreased excessive hepatic lipid accumulation. Simultaneously, serum AST and ALT levels in the PE-treated groups were reduced and liver protective effects were observed.

Some mechanisms associated with the anti-obesity effect of FX have been reported. The mechanisms of the anti-obesity effect are influenced by numerous factors, including nutritional, hormonal, and genetic elements [35]. The most well-known mechanism of the anti-obesity effect of FX is related to thermogenesis and lipolysis [36]. In our study, the anti-obesity effect of PE was evaluated in thermogenesis by examining UCP1 induction and lipolysis-related genes. UCP1 is mainly distributed in brown adipose tissue and acts in thermogenesis, controls energy expenditure, and protects against oxidative stress [37]. Additionally, UCP1 is activated by free fatty acids and is involved in the transport of hydrogen ions. This increase in UCP1 expression increases energy expenditure and thus is helpful for improving obesity. Additionally, the expression of genes related to WAT was evaluated to determine the effect of PE containing FX on adipogenesis. PPARγ and C/EBPα promote the proliferation and differentiation of mature adipocytes and are the most important transcription factors in adipogenesis [38]. Our results suggest that upregulation of UCP1 in WAT by PE containing FX significantly reduced abdominal and subcutaneous fat accumulation; the PE-M + CLA combination groups also showed increased UCP1 expression compared to the HFD group. The expression of PPARγ and C/EBPα was downregulated after administration of PE, contributing to the suppression of adipogenesis in WAT. Therefore, we concluded that the standardized PE derived from microalga PT exerted anti-obesity effects, with FX being the main functional compound. 

PE in combination with CLA appeared to have synergistic anti-obesity effects. The body weight- and body fat-lowering effects of CLA are controversial. CLA was reported to be effective for reducing weight and body fat in rats [39,40]. However, in hamsters, body fat was reduced but without weight loss [41]. Our results clearly demonstrated the increased anti-obesity effect of PE-M + CLA with respect to reduction in body weight, liver weight, adipose tissue weight, fat globules, fat volume, and the expression of proteins related to adipogenesis. Because FX is very hydrophobic, combining FX with other oils can further improve its transport through inter- and intracellular barriers, increasing its bioavailability to exert its effects. A previous study reported that the anti-obesity effects of FX were improved by combining it with medium-chain triacylglycerols; the adipose tissue weight gain was obviously lower and metabolic thermogenesis in the WAT was markedly increased in diabetic/obese KK-A^y^ mice fed a mixture of FX and medium-chain triacylglycerol oil [42]. Xanthigen is a well-known supplement used to control weight. This agent is a promising combination of FX from seaweed extract and pomegranate seed oil (an omega-5 long-chain polyunsaturated fatty acid). Xanthigen was shown to improve lipid metabolism in experimental and clinical studies [43]. In our study, a combination of PE and CLA synergistically enhanced the anti-obesity effect of FX, as well as its bioavailability and transport in the PE, suggesting the necessity to process or formulate PE with proper oils to increase the bioavailability of FX. 

## 4. Materials and Methods

### 4.1. Materials and Chemicals

*Phaeodactylum* extract (PE) used in this study was supplied by AlgaeTech Co., Ltd. (Gangneung, Republic of Korea). PE was in powder form and bright red in color and the amount of FX in PE was standardized to a range of 3.5~6% (*w/w*). The FX standard compound was purchased from Sigma–Aldrich (St. Louis, MO, USA). Dulbecco’s modified Eagle’s medium (DMEM), bovine calf serum, fetal bovine serum (FBS), penicillin/streptomycin, phosphate-buffered saline, insulin, and trypsin–EDTA were purchased from Gibco (Grand Island, NY, USA). Dexamethasone (DEX), 3-isobutyl-1-methylxanthine (IBMX), insulin, and Oil Red O were purchased from Sigma. All other chemicals were purchased from Sigma. 

### 4.2. HPLC Analysis

The HPLC analysis was performed with an Agilent 1260 HPLC system (Agilent Technologies, Santa Clara, CA, USA) with a CAPCELL PAK C18 MG II (5 μm particle size, 250 × 4.6 mm I.D.). The mobile phase consisted of acetonitrile and water with a flow rate of 1 mL/min. After loading the column with the PE in ethanol, the mobile phase was an acetonitrile: Water solution with the ratio increasing from 90:10 to 100:0 over 8 min, maintained at 100:0 for 3 min, and then decreased to 80:20 over 5 min. The absorption spectrum was obtained from 210 to 600 nm and the chromatogram was recorded at 450 nm. The FX standard was used for the construction of the calibration curve in the concentration range of 1~200 μg/mL.

### 4.3. Cell Cultures

3T3-L1 preadipocytes were cultured in DMEM containing 10% (*v/v*) bovine calf serum and 1% penicillin/streptomycin as antibiotics in a humidified atmosphere of 5% CO_2_ at 37 °C. The medium was changed every few days. When the cells were over 70% confluent, they were harvested via trypsinization and re-seeded into 25 cm^2^ culture flasks at a density of 3 × 10^3^ cells per cm^2^ in a fresh medium.

### 4.4. Cell Toxicity and Proliferation Assay

To evaluate cell cytotoxicity, 3T3-L1 cells (5 × 10^3^ cells/well) were cultured in a 96-well plate overnight. PE, FX, and curcumin were dissolved in dimethyl sulfoxide (DMSO) for cellular treatments. Cells were exposed or not exposed to the FX or PT extracts for 48 h. A cell toxicity assay was conducted using an EZ-Cytox cell viability assay kit (Daeil Lab Service, Seoul, Korea).

### 4.5. Cell Differentiation

Cells were seeded into a 6-well plate in DMEM containing 10% calf serum at a density of 3 × 10^4^ cells per well. The medium was changed every other day. Two-day post-confluent cells were stimulated for 2 days with a differentiation medium (DMEM containing 10% FBS, 0.5 mM IBMX, 1.0 μM DEX, and 1.0 μg/mL insulin). After 48 h, the medium was replaced with the adipocyte maintenance medium (DMEM containing 10% FBS and 10 μg/mL insulin) for 2 days. The cells were incubated with DMEM containing 10% FBS until day 8, when the cells should be fully differentiated. 3T3-L1 preadipocytes were treated with or without curcumin, FX, and PE starting on day 0 and maintained in the medium during the experiment.

### 4.6. Oil Red O Staining and Intracellular Triacylglycerol Level Measurements

The area of intracellular lipid accumulation was determined on day 6 by ORO staining. Briefly, the cells were fixed in 10% formaldehyde in phosphate-buffered saline for 1 h, and then washed with 60% isopropanol. The cells were stained with 0.5% ORO solution in 60:40 (*v/v*) isopropanol:distilled water (DW) for 20 min at room temperature, washed four times with water, and dried. Differentiation was quantified by dissolving the cells in isopropanol and measuring the optical density at 500 nm.

### 4.7. Animal Treatment

A total of 70e female C57BL/6J mice (4 weeks old) were purchased from Central Lab. Animal, Inc. (Seoul, Korea). All mice were housed in a room with controlled temperature (21 ± 2 °C), humidity (55 ± 15%), and lighting (12-h light/dark cycle). Mice had free access to water throughout the experiment. This study was approved by the Institutional Animal Care and Use Committee of Knotus (No. IACUC-18-KE-096).

After a 1-week adaptation period, the mice were randomly divided into the following seven groups (*n* = 10 per group): Normal diet (ND), high-fat diet (HFD), HFD plus FX 0.1 mg/kg/day, HFD plus PE 0.81 mg/kg/day (PE-L), HFD plus PE 1.62 mg/kg/day (PE-M), HFD plus PE 3.25 mg/kg/day (PE-H), and HFD plus PE 1.62 mg/kg/day groups in combination with a CLA 410 mg/kg/day (PE-M + CLA) group. Animals were fed with either a ND or HFD for 6 weeks ad libitum. The mice were given free access to food and water. For 6 weeks of feeding, FX and PE were suspended in distilled water by vortex mixing and sonication just before administration. Each suspension was administered orally at the volume of 10 mL/kg/day.

Body weight was measured twice a week for 6 weeks and an area under curve (AUC) was calculated using Graphpad Prism 5.03 software (San Diego, CA, USA) by one-way ANOVA. All data were expressed as mean ± standard error or mean ± standard deviation. At the end of the experiment period, the mice were fasted for 12 h and sacrificed. Blood was collected and organs (adipose tissues, liver) were rapidly removed, rinsed with physiological saline solution, weighed, and stored at −80 °C.

### 4.8. Serum Biochemical Measurements

The collected blood was incubated for 2 h at room temperature. Serum was prepared by centrifugation at 3500× *g* for 10 min at 4 °C and stored at −80 °C until use. The concentrations of triglyceride (TG), total cholesterol (TC), high-density lipoprotein (HDL), low-density lipoprotein (LDL), aspartate transaminase (AST), and alanine transaminase (ALT) were analyzed using a HITACHI 7180 chemistry analyzer (Tokyo, Japan) and enzyme-linked immunosorbent assay kit (R&D system, Inc., Minneapolis, MN, USA).

### 4.9. Histopathological Analysis

Adipose and liver pathological states were observed, and adipocyte size was quantified with a light microscope (Olympus BX53, Tokyo, Japan). Images were obtained using an image analyzer (Zen 2.3 blue edition, Carl Zeiss, Oberkochen, Germany).

### 4.10. Micro CT Analysis of Abdominal and Subcutaneous Fat Volume

The abdominal and subcutaneous fat in each mouse was scanned at an isotropic voxel size of 76 μm (45 kV, 177 μA, 200 ms integration time) with a viva CT 80 scanner (Scanco Medical, Brüttisellen, Switzerland). Two-dimensional gray-scale image slices were reconstructed by three-dimensional tomography. Density values for soft tissue were calibrated from a 5-point linear fit line with mixtures in various ratios of two liquids, ranging from 0.78 mg/mL (100% ethanol; Sigma) to 1.26 mg/mL (100% glycerol; J.T. Baker, Phillipsburg, NJ, USA). The measured body fat area was the abdominal fat and subcutaneous fat in the space from the second lumbar spine to the fifth lumbar vertebra. All animals were imaged using the standard micro-CT imaging protocol (220 views, 16 ms X-ray exposure time, and 70 kV/32 mA penetration energy). Reconstruction of micro-CT images was performed using MicroView (GE Healthcare, Little Chalfont, UK) software packages.

### 4.11. Western Blot Analysis

The cells or tissues were lysed in a cold RIPA buffer (pH 7.4) containing 10 μM phenylmethanesulfonyl fluoride and a 1% protease inhibitor cocktail. Cells and tissues lysates were centrifuged at 13,000 rpm for 10 min at 4 °C. The protein contents were analyzed via the BCA (bicinchoninic acid) protein assay kit (Vazyme Biotech Co., Ltd., Nanjing, China). Equal quantities of protein were electrophoresed by sodium dodecyl sulfate-polyacrylamide gel electrophoresis and transferred to polyvinylidene fluoride membranes (Millipore, Billerica, MA, USA). Membranes were incubated with antibodies including those for UCP1, PPARγ, and C/EBPα at 4 °C overnight. β-Actin was used as a control with a β-actin antibody (Santa Cruz Biotechnology, Dallas, TX, USA). The protein markers were visualized via chemiluminescence (Western Lighting Plus-ECL, PerkinElmer, Waltham, MA) and with a Chemidoc image analyzer (Bio-Rad, Hercules, CA, USA).

### 4.12. Statistical Analysis

Data were analyzed using the GraphPad Prism 5.03 software (San Diego, CA, USA). All data were expressed as the mean ± standard error or mean ± standard deviation. Statistical significances between groups were determined by one-way analysis of variance, followed by Dunnett’s multiple tests. A value of *p <* 0.05 represents a significant difference.

## 5. Conclusions

In conclusion, PE significantly reduced body weight gain and adipose tissue weight by activating UCP1 and inactivating PPARγ and C/EBPα. Moreover, PE remarkably reduced fat mass in HFD-induced mice. The efficacy of PE was equal to that of the positive control substance, FX, and was potentiated when combined with CLA. Our results indicate that PE containing FX exerts anti-obesity effects by promoting lipolysis and inhibiting lipogenesis and is a good candidate for the development of anti-obesity foods and health functional foods derived from new marine microalgae.

## Figures and Tables

**Table 1:** Effects of PE on serum parameters in the plasma.

	ND	HFD	FX	PE-L	PE-M	PE-H	PE-M + CLA
**Plasma**							
TG (mg/dL)	45.3 ± 10.3	50.6 ± 9.2	44.7 ± 17.2	47.6 ± 10.2	34.8 ± 13.5 ^#^	35.7 ± 18.2	31.3 ± 6.7 ^#^
TC (mg/dL)	79.5 ± 5.8	130.5 ± 6.1 ***	128.2 ± 30.9 ***	126.3 ± 20.3 ***	137.2 ± 9.2 ***	123.7 ± 18.4 ***	133.1 ± 14.3 ***
HDL (mg/dL)	48.1 ± 6.0	71.2 ± 4.0 ***	63.8 ± 15.7 **	63.3 ± 11.9 **	68.2 ± 5.6 ***	60.9 ± 10.8 *	64.3 ± 7.8 **
LDL (mg/dL)	6.8 ± 1.0	17.2 ± 1.6 ***	17.3 ± 5.3 ***	15.7 ± 1.7 ***	14.9 ± 1.9 ***	14.1 ± 1.7 ***^,#^	14.4 ± 1.8 ***
AST (U/L)	30.8 ± 4.2	31.5 ± 10.6 *	23.1 ± 4.8	23.4 ± 6.1	25.0 ± 3.2	26.8 ± 9.1	22.3 ± 8.0
ALT (U/L)	52.7 ± 6.6	64.7 ± 10.9	61.4 ± 12.8 ^#^	55.1 ± 7.1	56.1 ± 4.5	62.5 ± 10.5	55.8 ± 10.7 *^,#^

Note: Data are expressed as the mean ± SD (*n* = 10 per group). Triacylglyceride (TG); total cholesterol (TC); high-density lipoprotein (HDL); low-density lipoprotein (LDL);aspartate transaminase (AST); alanine transaminase (ALT); normal diet (ND); high fat diet (HFD); fucoxanthin (FX), HFD + FX 0.1 mg/kg/day; PE-L, HFD + PE 0.81 mg/kg/day; PE-M, HFD + PE 1.62 mg/kg/day; PE-H, HFD + PE 3.25 mg/kg/day; PE-M + CLA, HFD + PE 1.62 mg/kg/day plus CLA 410 mg/kg/day. ***/**/* indicate a significant difference at *p <* 0.001/*p*< 0.01/*p*< 0.05 levels compared to the ND. # indicates a significant difference at *p <* 0.05 level compared to the HFD.

## Supplementary Materials

The following are available online at https://www.mdpi.com/1660-3397/17/5/311/s1, Figure S1: HPLC chromatogram of FX standard and PE recorded at 450 nm, Figure S2: Cytotoxicity of fucoxanthin, PE and curcumin toward 3T3-L1 cells, Table S1: The average food intake (g/day) contents of C57BL/6J mice.

## References

[1] D’Orazio N., Gemello E., Gammone M.A., de Girolamo M., Ficoneri C., Riccioni G. (2012). Fucoxanthin: A treasure from the sea. Mar. Drugs.

[2] Kim S.M., Jung Y.J., Kwon O.N., Cha K.H., Um B.H., Chung D.H., Pan C.H. (2012). A potential commercial source of fucoxanthin extracted from the microalga *Phaeodactylum tricornutum*.. Appl. Biochem. Biotechnol..

[3] Kim S.M., Kang S.W., Kwon O.N., Chung D.W., Pan C.H. (2012). Fucoxanthin as a major carotenoid in *Isochrysis aff. galbana*: Characterization of extraction for commercial application. J. Korean Soc. Appl. Biol. Chem..

[4] Xia S., Wang K., Wan L., Li A., Hu Q., Zhang C. (2013). Production, characterization, and antioxidant activity of fucoxanthin from the marine diatom *Odontella aurita*.. Mar. Drugs..

[5] Lee S.H., Min K.H., Han J.S., Lee D.H., Park D.B., Jung W.K., Park P.J., Jeon B.T., Kim S.K., Jeon Y.J. (2012). Effects of Brown Algae, *Ecklonia cava* on Glucose and Lipid Metabolism in C57BL/KsJ-db/db Mice, a Model of Type 2 Diabetes mellitus. Food Chem. Toxicol..

[6] Okada T., Mizuno Y., Sibayama S., Hosokawa M., Miyashita K. (2011). Antiobesity Effects of *Undaria* Lipid Capsules Prepared with Scallop Phospholipids. J. Food Sci..

[7] Fung A., Hamid N., Lu J. (2013). Fucoxanthin Content and Antioxidant Properties of *Undaria pinnatifida*.. Food Chem..

[8] Jeon S.M., Kim H.J., Woo M.N., Lee M.K., Shin Y.C., Park Y.B., Choi M.S. (2010). Fucoxanthin-rich seaweed extract suppresses body weight gain and improves lipid metabolism in high-fat-fed C57BL/6J mice. Biotechnol. J..

[9] Woo M.N., Jeon S.M., Kim H.J., Lee M.K., Shin S.K., Shin Y.C., Park Y.B., Choi M.S. (2010). Fucoxanthin supplementation improves plasma and hepatic lipid metabolism and blood glucose concentration in high-fat fed C57BL/6N mice. Chem. Biol. Interact..

[10] Gammone M.A., D’Orazio N. (2015). Anti-Obesity Activity of the Marine Carotenoid Fucoxanthin. Mar. Drugs.

[11] Beppu F., Hosokawa M., Niwano Y., Miyashita K. (2012). Effects of dietary fucoxanthin on cholesterol metabolism in diabetic/obese KK-A(y) mice. Lipids Health Dis..

[12] Rangwala S.M., Lazar M.A. (2000). Transcriptional control of adipogenesis. Annu. Rev. Nutr..

[13] Tong L. (2005). Acetyl-coenzyme A carboxylase: Crucial metabolic enzyme and attractive target for drug delivery. Cell. Mol. Life Sci..

[14] Maeda H., Hosokawa M., Sashima T., Murakami-Funayama K., Miyashita K. (2009). Anti-obesity and anti-diabetic effects of fucoxanthin on diet-induced obesity conditions in a murine model. Mol. Med. Rep..

[15] Woo M.N., Jeon S.M., Shin Y.C., Lee M.K., Kang M.A., Choi M.S. (2009). Anti-obese property of fucoxanthin is partly mediated by altering lipid-regulating enzymes and uncoupling proteins of visceral adipose tissue in mice. Mol. Nutr. Food Res..

[16] Rebello C.J., Greenway F.L., Johnson W.D., Ribnicky D., Poulev A., Stadler K., Coulter A.A. (2017). Fucoxanthin and its metabolite fucoxanthinol do not induce browning in human adipocytes. J. Agric. Food Chem..

[17] Hu X., Tao N., Wang X., Xiao J., Wang M. (2016). Marine-derived bioactive compounds with anti-obesity effect: A review. J. Func. Foods.

[18] Miyashita K., Hosokawa M. (2017). Fucoxanthin in the management of obesity and its related disorders. J. Func. Foods.

[19] Muradian K.H., Vaiserman A., Min K.J., Frafield V.E. (2015). Fucoxanthin and lipid metabolism: A mini review. Nutr. Metab. Cardiovasc. Dis..

[20] Gautron L., Elmquist J.K. (2011). Sixteen years and counting: An update on leptin in energy balance. J. Clin. Invest..

[21] Roujeau C., Jockers R., Dam J. (2014). New pharmacological perspectives for the leptin receptor in the treatment of obesity. Front. Endocrinol..

[22] Heilbronn L.K., Noakes M., Clifton M.P. (2001). Energy restriction and weight loss on very-low-fat diets reduce C-reactive protein concentrations in obese, healthy women. Atheroscler. Thromb. Vasc. Biol..

[23] Maeda H., Tsukui T., Sashima T., Hosokawa M., Miyashita K. (2008). Seaweed carotenoid, fucoxanthin, as a multi-functional nutrient. Asia Pac. J. Clin. Nutr..

[24] Kim J.H., Kim S.M., Cha K.H., Mok I.K., Koo S.I., Pan C.H., Lee J.K. (2016). Evaluation of the anti-obesity effect of the microalga *Pheodactylum tricornutum*.. Appl. Biol. Chem..

[25] Kang S.I., Ko H.C., Shin H.S., Kim H.M., Hong Y.S., Lee N.H., Kim S.J. (2011). Fucoxanthin exerts differing effects on 3T3-L1 cells according to differentiation stage and inhibits glucose uptake in mature adipocytes. Biochem. Biophys. Res. Commun..

[26] Wu Z., Rosen E.D., Brun R., Hauser S., Adelmant G., Troy A.E., McKeon C., Darlington G.J., Spiegelman B.M. (1999). Cross-Regulation of C/EBPα and PPARγ Controls the Transcriptional Pathway of Adipogenesis and Insulin Sensitivity. Mol. Cell..

[27] Kim E.H., Bae J.S., Hahm K.B., Cha J.Y. (2012). Endogenously synthesized n-3 polyunsaturated fatty acids in *fat-1* mice ameliorate high-fat diet-induced non-alcoholic fatty liver disease. Biochem. Pharmacol..

[28] Fuster J.J., Castillo A.I., Zaragoza C., Ibáňez B., Andrés V. (2012). Animal models of atherosclerosis. Prog. Mol. Biol. Transl. Sci..

[29] Park S., Kim K., Han S.I., Kim E.J., Choi Y.E. (2017). Organic solvent-free lipid extraction from wet *Aurantiochytrium* sp. Biomass for co-production of biodiesel and value-added products. Appl. Biol. Chem..

[30] Kumar V., Kumar R., Rawat D., Nanda M. (2018). Synergistic dynamics of light, photoperiod and chemical stimulants influences biomass and lipid productivity in *Chlorella singularis* (UUIND5) for biodiesel production. Appl. Biol. Chem..

[31] Conde E., Moure A., Domíngues H. (2015). Supercritial CO_2_ extraction of fatty acids, phenolics and fucoxanthin from free-dried *Sargassum muticum*.. J. Appl. Phycol..

[32] Kanazawa K., Ozaki Y., Hashimoto T., Das S.K., Mastushita S., Hirono M., Okada T., Komoto A., Mori N., Kakatsuka M. (2008). Commrecial-scale preparation of biofunctional fucoxanthin from waste parts of brown sea algae *Laminalia japonica*.. Food Sci. Technol. Res..

[33] Ambati R., Phang S.M., Ravi S., Aswathanarayana R. (2014). Astaxanthin: Source, extraction, stability, biological activities and its commercial applications-a review. Mar. Drugs.

[34] Renella M.E. (2015). Nonalcoholic fatty liver disease: A systematic review. JAMA.

[35] Pan H., Fu C., Huang L., Jiang Y., Deng X., Guo J., Su Z. (2018). Anti-obesity effect of chitosan oligosaccharide capsules (COSCs) in obese rats by ameliorating leptin resistance and adipogenesis. Mar. Drugs.

[36] Maeda H., Hosokawa M., Sashima T., Funayama K., Miyashita K. (2005). Fucoxanthin from edible seaweed *Undaria Pinnatifida*, shows anti-obesity effect through UCP1 expression in white adipose tissues. Biochem. Biophys. Res. Commun..

[37] Maeda H. (2015). Nutraceutical effects of fucoxanthin for obesity and diabetes therapy: A review. J. Oleo Sci..

[38] Ali A.T., Hochfeld W.E., Myburgh R., Pepper M.S. (2013). Adipocyte and adipogenesis. Eur. J. Cell. Biol..

[39] Hu X., Li Y., Li C., Fu Y., Cai F., Chen Q., Li D. (2012). Combination of fucoxanthin and conjugated linoleic acid attenuates body weight gain and improves lipid metabolism in high-fat diet-induced obese rats. Arch. Biochem. Biophys..

[40] Banni S., Carta G., Angioni E., Murru E., Scanu P., Melis M.P., Bauman D.E., Fischer S.M., Ip C. (2001). Distribution of conjugated linoleic acid and metabolites in different lipid fractions in the rat liver. J. Lipid Res..

[41] Navarro V., Zabala A., Macarulla M.T., Fernández-Quintela A., Rodríguez V.M., Simón E., Portillo M.P. (2003). Effects of conjugated linoleic acid on body fat accumulation and serum lipids in hamsters fed an astherogenic diet. J. Physiol. Biochem..

[42] Maeda H., Hosokawa M., Sashima T., Miyashita K. (2007). Dietary combination of fucoxanthin and fish oil attenuates the weight gain of white adipose tissue and decrease blood glucose in obese/diabetic KK-A^y^ mice. J. Agric. Food Chem..

[43] Abidov M., Ramazanov Z., Seifulla R., Grachev S. (2010). The effect of Xanthigen in the weight management of obese premenopausal women with non-alcoholic fatty liver disease and normal liver fat. Diabetes Obes. Metab..

