# Supplementary material for: Anti-Obesity Effect of Standardized Extract of Microalga Phaeodactylum tricornutum Containing Fucoxanthin
Source: Mar Drugs. 2019 May 27;17(5):311. doi: 10.3390/md17050311 (PMC6562887; doi:10.3390/md17050311)
Supplement: Supplementary file 1 [file marinedrugs-17-00311-s001.pdf]

Supplementary Materials:

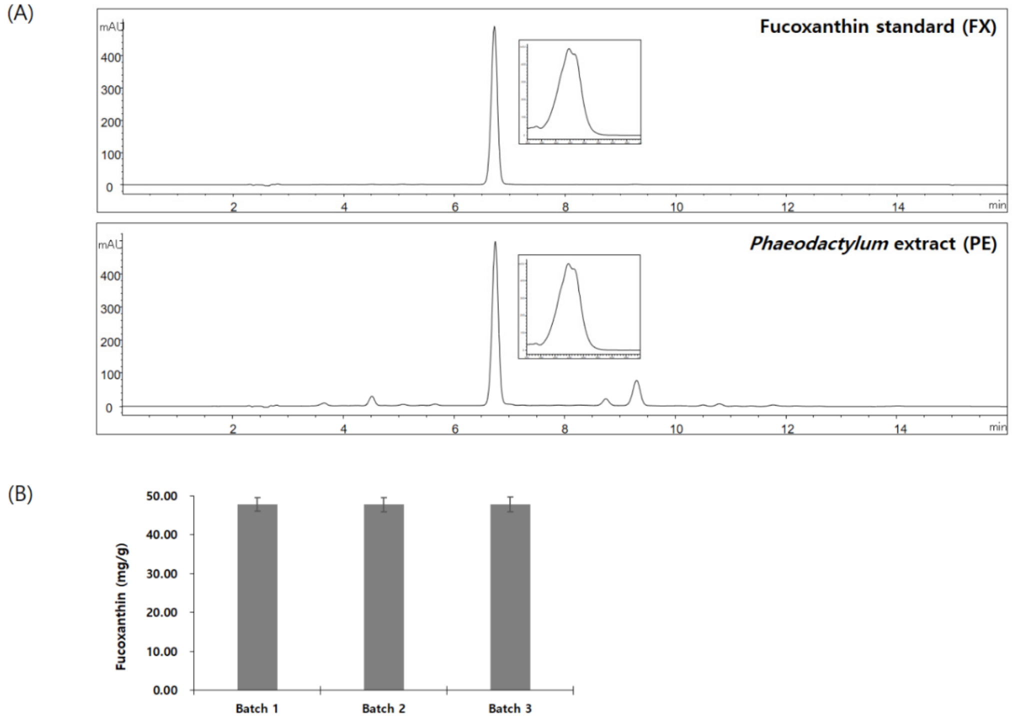

Figure S1. HPLC chromatogram of FX standard (A) and PE (B) recorded at 450 nm. Absorption spectrum of FX peak was obtained from 210 to 600 nm. FX content was quantified from three batches of PE and the average of three batches was 47.74 ± 0.04 mg/g. There was no significant difference among three batches at $p < 0.05$.

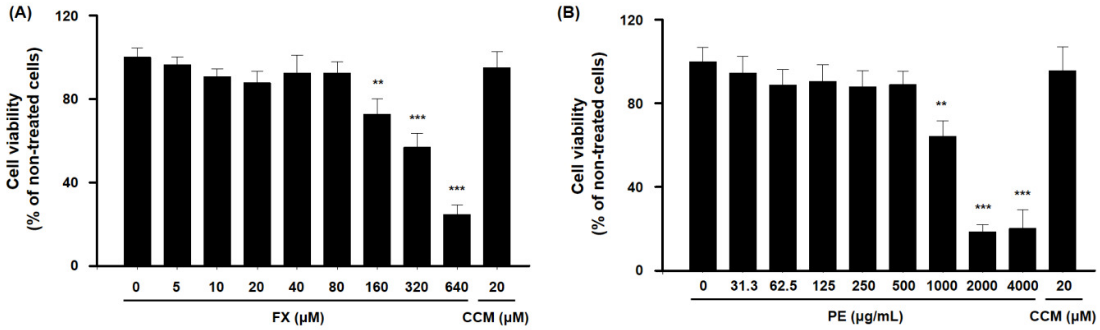

Figure S2. Cytotoxicity of fucoxanthin, PE, and curcumin (CCM; reference) toward 3T3-L1 cells. Viability of cells treated for 48 h with fucoxanthin, PE, and CCM. Absorbance at 450 and 650 nm was recorded using an ELISA plate reader. (**A**) Fucoxanthin and CCM; (**B**) PE and CCM. ***/** indicate significant differences at $p < 0.001$/$p < 0.01$ levels compared with the untreated group. Values are shown as the mean ± SD (n = 6 per group).

Table 1. The average food intake (g/day) contents of C57BL/6J mice.

	Group						
	ND	HFD	FX	PE-L	PE-M	PE-H	PE-M + CLA
Average food intake (g/day)	3.55 ± 0.31	3.46 ± 0.25	3.26 ± 0.09	3.27 ± 0.19*	3.31 ± 0.16	3.29 ± 0.15	3.18 ± 0.17

* indicate significant differences at p < 0.05.
